# Software Reference Architecture for Real-Time Mobile Digital Phenotyping: Evaluation of System Designs

**DOI:** 10.2196/87320

**Published:** 2026-08-07

**Authors:** Ian Kim, Thomas N Robinson, Byron B Reeves, Nick Haber, Nilàm Ram

**Affiliations:** 1 Department of Pediatrics, Stanford Medicine, Stanford, CA, United States; 2 Department of Psychology, Stanford University, 450 Jane Stanford WayStanford, CA, 94305, United States, 1 6507232300; 3 Department of Medicine, Stanford Medicine, Stanford, CA, United States; 4 Department of Communication, Stanford University, Stanford, CA, United States; 5 Graduate School of Education, Stanford University, Stanford, CA, United States

**Keywords:** mobile computing, edge computing, computer architecture, systems engineering, multimodal data fusion, mobile health, digital phenotyping, Screenomics, mobile phone

## Abstract

**Background:**

Digital phenotyping—the use of continuous data streams from digital devices such as smartphones to assess behavioral, psychological, and physiological states—holds transformative potential for health monitoring and personalized care. However, real-time analysis of large multimodal data often exceeds mobile devices’ computational resources, leading most platforms to rely on sequential processing and cloud-based computation.

**Objective:**

We propose the Stanford Screenomics platform as a software reference architecture that uses a modular design to integrate parallel processing and edge computing, enabling scalable, real-time digital phenotyping on smartphones.

**Methods:**

Two prototype apps were developed: one following the parallel, on-device architecture (Stanford Screenomics platform) and another based on a traditional sequential, cloud-based design (traditional). Both processed identical multimodal data streams at the same intensity; only the location and sequence of computation differed. In two 48-hour experiments, performances were compared across four load profiles: low (≈10 MB/min), medium (≈30 MB/min), heavy (≈40 MB/min), and very heavy (≈60 MB/min). In the first experiment, offline resource performance was assessed under continuous simulated smartphone use. Virtual users completed six tasks in a fixed five-minute sequence: watching YouTube (Google LLC), reading eBooks, browsing TikTok (ByteDance Ltd), web surfing, listening to Spotify, and scrolling Instagram Reels (Meta). Minute-by-minute measurements of CPU usage (%), RAM usage (MB), battery drain (%/h), and data loss (%) were collected. Descriptive statistics (mean±SD) summarized performance, and independent *t* tests compared architectures. Data loss trajectories were analyzed to determine whether growth was linear or exponential under increasing load. In the second experiment, end-to-end phenotyping latency was evaluated over stable Wi-Fi. Five key-stage timestamps per trial tracked local writes, preprocessing, memory parsing, phenotype analysis, and intervention delivery. Total phenotype update time per trial was the primary outcome, and latency differences between architectures were analyzed using linear mixed-effects models, with IQRs reported to capture variability across load conditions.

**Results:**

The Stanford Screenomics platform consistently demonstrated lower CPU usage (3.9%‐14.6% vs 10.5%‐26.9%) and RAM usage (97‐132  MB vs 101‐155  MB) than the traditional, with reduced battery drain (0.9%‐2.1%/h vs 1.4%‐3.2%/h). Data fidelity was higher in the Stanford Screenomics, with shallow linear data loss (0.4%‐1.5%/h) compared to exponential growth in the traditional (2%‐7.1%/h), achieving up to 9.4× greater data retention under very heavy load. The Stanford Screenomics completed phenotype updates in 0.90 seconds under low load and 9.32 seconds under very heavy load, compared to 30.1‐398.1  seconds for traditional, representing 34‐43×faster processing with substantially narrower variability (IQR 0.3‐6 s vs 11  s-5  min).

**Conclusions:**

These results demonstrate that the Stanford Screenomics platform architecture enables real-time, on-device digital phenotyping with high fidelity and low latency. This validated prototype architecture establishes a resilient foundation for the next generation of scalable, reliable, and context-aware deployment of real-world mobile health interventions on mobile devices.

## Introduction

### Digital Phenotyping: Opportunities and Applications

Digital phenotyping is an innovative health monitoring method that involves collecting and analyzing longitudinal digital trace data to characterize individuals’ behavioral, psychological, and physiological states in real-world contexts [1]. This approach aims to provide a detailed and timely understanding of individual health as it naturally unfolds in everyday life. Smartphones are particularly well-suited for digital phenotyping due to their ubiquity, portability, and rich array of built-in sensors that enable real-time, continuous capture of diverse, high-frequency data streams. Complementary to electronic health records or web browsing histories, smartphone-based traces provide fine-grained insights into individual-specific dynamics across time and space, including mobility patterns, interaction behaviors, and media exposure. Prior studies have shown the predictive potential of these smartphone-based signals; for instance, using physical activity data and insulin injection logs to forecast hypoglycemic events in individuals with diabetes [2,3], and using screen activity to detect early signs of mental health crises [4]. As demonstrated in these studies, smartphone-based digital phenotyping holds significant promise as a foundation for delivering timely, adaptive medical and behavioral interventions that support individuals’ health.

### Challenges in Mobile Digital Phenotyping

Identifying and tracking the evolution of individuals’ health phenotypes requires sustained collection of continuous, real-time data from multimodal sensors and concurrent analysis of heterogeneous data streams. This poses significant engineering challenges due to the limited processing power, memory, and storage of smartphones [5-7]. These hardware constraints generally preclude the use of large-scale multiple-input or multiple-output models or extensive modality-specific preprocessing that would otherwise support interoperability across diverse data modalities. Consequently, most existing digital phenotyping systems adopt a sequential, cloud-centric processing model, in which data are collected on-device, processed in a linear task order, and periodically offloaded to external servers for retrospective analysis. This “traditional” model—defined here as sequential on-device ingestion with delayed cloud-based processing—constitutes the dominant architectural baseline in widely used open-source mobile sensing platforms, including Beiwe and AWARE, where raw sensor streams are written locally and batch-uploaded for offline feature extraction [8,9]. This sequential, cloud-centric pipeline has two key bottlenecks: a throughput bottleneck, where sequential processing limits data handled per unit time, and overall performance is constrained by the slowest task [10,11], and a bandwidth bottleneck, where data transmission capacity delays off-device processing [12,13]. When mobile resources (ie, CPU and memory) are strained by these bottlenecks, a third constraint, the power wall, emerges, wherein thermal and energy limitations prevent processors from scaling performance [14-16]. Hitting this power wall results in further performance degradation, heightened data loss, and an increased risk of OS app suspension. Ultimately, the constraints persistently threaten to interrupt ongoing data collection and introduce significant blind spots into longitudinal health records. In short, the promise of smartphone-based digital phenotyping and delivery of timely, adaptive medical and behavioral interventions that support individuals’ health cannot be realized if systems continue to rely on the bottleneck-prone sequential, cloud-centric architecture on which the current systems are built. Emerging applications—including just-in-time adaptive interventions, relapse prevention, acute stress detection, and context-aware behavioral coaching—require new architectures that can generate accurate and timely digital phenotypes. It is increasingly critical, for both patient safety and treatment efficacy, that we develop new architectures that are immune to network latency, data loss, and service interruptions.

### Solutions: Parallel Processing, Edge Computing, and Modular Architecture

To address the engineering challenges noted above, mobile systems researchers are increasingly exploring parallel processing [7,17,18] and edge computing architectures [13,19,20]. While these approaches have been successfully applied in domains such as consumer on-device AI, robotics, and social media personalization [21-26], where high data throughput and real-time responsiveness are essential, they have not been integrated into mHealth (mobile health) tools. We propose here how these approaches might be successfully leveraged into a new architecture to support continuous, real-time digital phenotyping and delivery of timely, context-sensitive interventions that better help individuals achieve their health goals.

Parallel processing accelerates task execution by dividing computation into smaller fragments that run simultaneously across multiple processor cores. This kind of processing is particularly effective for tasks that involve heavy computation, have strict time constraints, or can be divided into smaller subtasks. For example, in video recording with real-time filters, one core may handle video capture, while another performs image stabilization, and a third applies color correction or compression. In mHealth, the parallel idea would be to concurrently process physiological signals on one core, behavioral indicators on another, and contextual information on a third, enabling richer multimodal inputs that improve predictive performance for downstream digital phenotyping and health-related event detection.

Implementing parallel processing in mHealth tools requires sophisticated task scheduling. Beyond minimizing total completion time, scheduling must ensure that time-sensitive computations (eg, crisis-detection alerts) take precedence over lower-priority tasks. Developers often rely on the OS-level thread schedulers due to the complexity of custom scheduling and variability in hardware capabilities across devices, including differences in core architectures and memory bandwidth. However, OS-managed scheduling has key limitations: limited developer control over timing and execution order, potential disregard for task priorities or dependencies, and unpredictable delays introduced by system-wide multitasking. Concurrency debugging is also more difficult when execution is dynamically determined by the OS. Health-related priorities may get downgraded by OS-level schedulers.

Manual task scheduling allows explicit control over task priorities, dependencies, and computational load when determining execution order and timing. A manual scheduler can manage queues, allocate resources, and adapt execution based on system load, battery status, task progress, and/or clinical priority. However, it is difficult to implement due to the need for variable execution times, intermodule coordination, and constrained CPU, memory, and battery resources. A common compromise is heuristic scheduling, which approximates near-optimal task ordering for complex workflows. In the hybrid approach, the OS manages low-level thread execution while a higher-level platform scheduler coordinates tasks, prioritizes critical processes, and optimizes resource use. When combined with modular design—where task-specific modules internally track dependencies and computational load—heuristic scheduling can outperform both fully OS-driven and fully manual scheduling in efficiency and reliability. By dividing intensive workloads into subtasks and coordinating their uninterrupted execution through hybrid scheduling, intelligent parallel processing can enable the handling of more complex and fine-grained multimodal data streams, improving information fidelity to support more accurate health event detection.

### Continuous, Priority-Aware Digital Phenotyping With Real-Time Decisioning Under Resource Constraints

Edge computing refers to performing data processing, storage, and analysis directly on the device where data are generated, rather than transmitting raw data to centralized servers [13,27]. For example, modern smartphones can perform on-device speech recognition, converting audio to text locally without cloud infrastructure. By keeping computation at the data source, edge computing reduces latency, lowers bandwidth consumption, and minimizes energy costs associated with continuous network communication.

Despite its potential to complement parallel processing and further improve performance, edge computing is often constrained by limited mobile hardware resources. Unlike cloud servers that scale elastically on demand, on-device computation requires careful preallocation of CPU and memory. Overestimation can trigger OS-level interventions such as thermal throttling or application termination. These constraints become more pronounced when synchronizing fragmented data streams generated through parallel processing, which must be merged into a coherent local state for joint analysis. Ensuring efficient synchronization without exceeding CPU or memory limits is significantly more complex than offloading coordination to centralized cloud servers.

Recent advances in mobile systems, however, suggest that architectural strategies enabling more efficient resource allocation might make edge computing more feasible. One approach is software decomposition into micromodules tailored to specific hardware targets [13,28,29]. This modular design is often paired with a reactive resource manager that monitors system telemetry—thermal state, battery level, and available memory—and dynamically adjusts execution. For instance, if a device approaches thermal limits, the manager may reduce parallelism or defer tasks to a single-threaded queue to prevent throttling [30-32]. Replacing centralized on-device storage with a logically distributed storage layer further improves efficiency by enabling modules to share processed outputs directly [33,34]. In-memory caching is central to this design: intermediate results are stored in RAM rather than disk, enabling reuse without redundant computation. By minimizing data movement between storage and processing units, in-memory caching reduces latency, alleviates bandwidth bottlenecks, and lowers energy consumption during continuous multimodule execution. Coupling edge computing with a reactive resource manager, therefore, can enable continuous on-device, parallel processing of rich multimodal data under constrained resources, advancing mHealth toward always-on and low-latency digital phenotyping and intervention delivery.

Together, with careful implementation, mobile apps that require both high computational throughput and real-time responsiveness can benefit substantially from combining parallel processing and edge computing within a modular architecture. In such systems, task-specific modules manage their own CPU, memory, and storage demands, while a reactive resource manager dynamically balances system load based on battery, thermal, and memory conditions and informs modules’ scheduling decisions. Strategies such as asynchronous task pipelines, distributed storage across modules, and data caching reduce throughput and bandwidth bottlenecks that would otherwise arise from sequential cloud processing. By integrating careful task scheduling with local computation and adaptive resource management, these architectures can exploit the speed advantages of parallelism while minimizing energy costs and addressing the power wall limitations. In turn, systems with these architectures can handle heavier data loads with improved energy efficiency, greater data fidelity, and more stable operation over extended periods [5,20,35-37]. The gains in efficiency and reliability of real-time processing should, in principle, enable faster phenotype detection and timelier adaptive mHealth interventions, which are critical for real-world deployment.

### Software Reference Architecture for Real-Time Mobile Digital Phenotyping: The Stanford Screenomics Platform

There has been some effort in mobile digital phenotyping to improve efficiency within existing system constraints via task-level optimizations such as adaptive sensor sampling, energy-aware scheduling, and simplified analytical models [1,38]. While these approaches mitigate some performance limitations, they cannot fully overcome fundamental throughput and memory constraints inherent in high-frequency multimodal data collection on resource-constrained smartphones. Here, we propose, implement, and evaluate a new software architecture that integrates parallel processing with edge computing to enable real-time mobile digital phenotyping through modular systems design. As these system-level advances were not integrated into the currently used mHealth frameworks, the conventional smartphone data processing architecture used in most digital health apps requires a fundamental redesign. We thus establish the Stanford Screenomics platform as the first software reference architecture designed to support clinically responsive, real-time mobile digital phenotyping.

**Figure 1.**
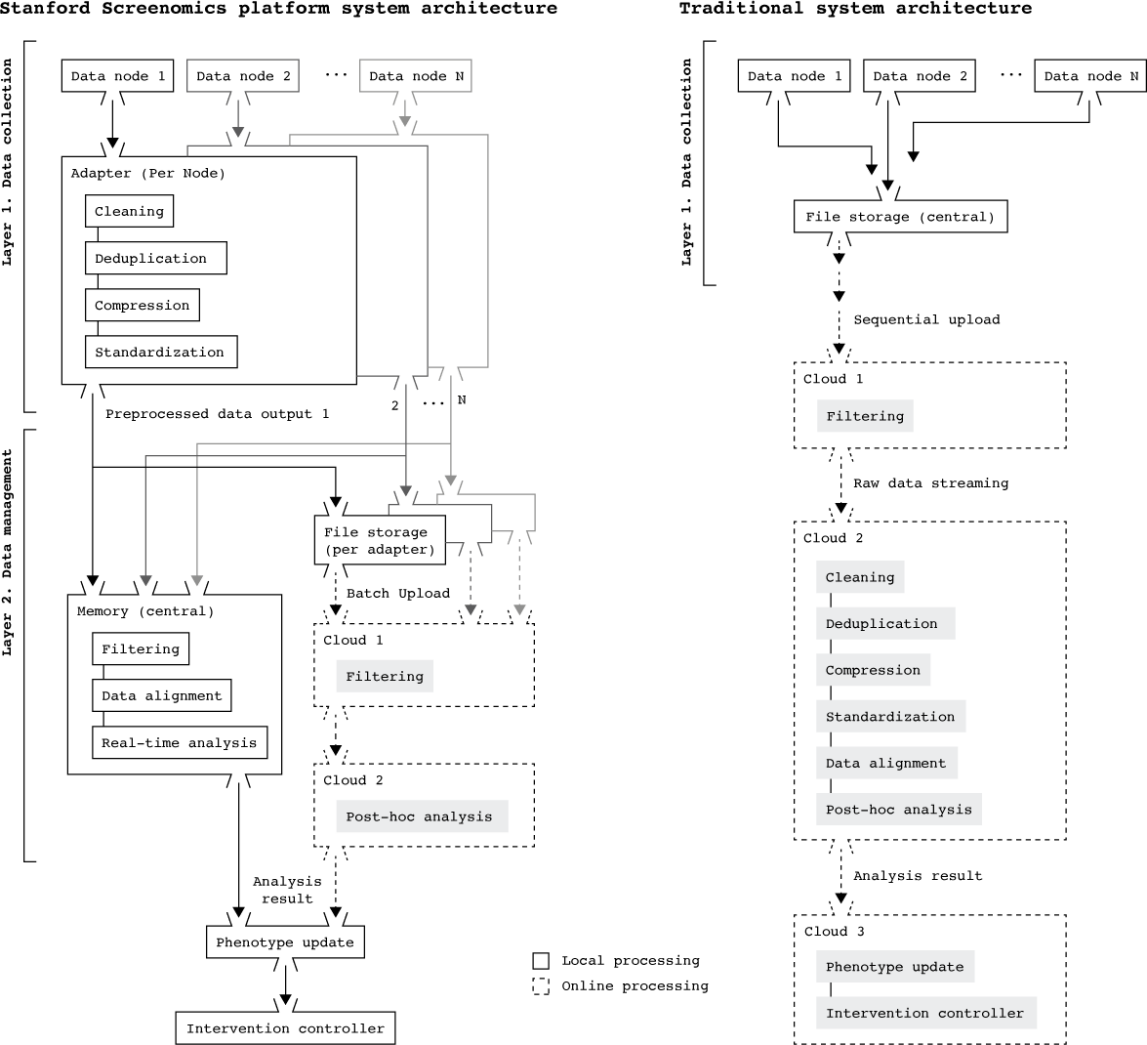
Comparison of key workflow steps in the Stanford Screenomics platform system architecture and traditional system architecture, from data collection through to intervention decision-making.

The key novelty lies in the architectural integration of parallel processing and edge computing through modular decomposition, in which each module processes a given data source independently and in parallel, supported by distributed storage and in-memory caching to enable localized on-device computation. A reactive resource manager dynamically schedules asynchronous task pipelines across modules based on device conditions, while a custom multimodal data fusion pipeline performs early-phase data standardization, transforming heterogeneous inputs through modality-specific preprocessing into a unified format for cross-module compatibility. Figure 1 illustrates the structural shift from traditional sequential, cloud-based models to the Stanford Screenomics platform reference architecture, specifically highlighting the embedded data processing layers and the dual engines driving automated care: the phenotype engine and the intervention engine.

While traditional architectures have successfully enabled retrospective digital phenotyping via delayed cloud-based analytics—such as predicting schizophrenia relapse or estimating addiction cravings—their sequential pipelines introduce substantial latencies ranging from minutes to days [39,40]. The primary aim of this study is to formally describe and evaluate the proposed parallel, on-device architecture relative to a traditional sequential, cloud-based phenotyping system that defines the field’s current data-ingestion status quo. We hypothesize that shifting computation to the edge and enabling modular parallel execution would significantly improve system efficiency and performance, demonstrating its potential to enable a robust, scalable, and real-time digital phenotyping to safely support automated clinical interventions in the wild.

## Methods

### Study Design

To evaluate real-time mobile digital phenotyping performance, we developed and compared two system prototypes: the Stanford Screenomics platform system architecture and a traditional system architecture representing the prevailing sequential, cloud-based processing model (Figure 1). The Stanford Screenomics platform is a modular, edge-centric architecture consisting of a data collection layer using a unified fusion standard, a distributed data management layer leveraging distributed storage, a phenotype engine, and an intervention engine. Parallel processing, asynchronous task scheduling, and in-memory caching enable localized, real-time phenotype generation while dynamically balancing computational load. In contrast, the traditional system architecture uses a sequential processing pipeline in which raw data streams are written to local storage and periodically batch-uploaded to a centralized server for retrospective feature extraction. Detailed technical specifications for both architectures are provided in Multimedia Appendix 1.

We conducted two 48-hour real-time health monitoring experiments, using the two system prototypes and architectures, to evaluate system performance across four system-level benchmarks: resource usage, energy efficiency, data fidelity, and processing speed. Resource usage was measured using continuous CPU and RAM consumption, energy efficiency by battery consumption, data fidelity by the completeness and integrity of collected data streams, and processing speed by throughput and execution latency. These benchmarks determine the feasibility of sustained real-time digital phenotyping on resource-constrained mobile devices. To support real-time health interventions, a mobile platform must optimize these metrics to sustain stable, continuous background monitoring while simultaneously delivering rapid and adaptive responsiveness, required for real-time health interventions, thereby preventing clinical blind spots and intervention decay [8,38]. By evaluating both architectures across these dimensions under varying data-load conditions, we isolated the impact of architectural design choices on the technical viability of real-time mobile digital phenotyping.

### System Implementation

To evaluate performance differences between architectural designs, we implemented both the Stanford Screenomics platform architecture—a distributed, parallel in-memory processing pipeline—and the traditional architecture—a centralized, cloud-dependent sequential pipeline. Each of these Android apps was developed in Android Studio and integrated with Firebase (Google LLC) services—including Firestore, Cloud Functions, and Cloud Storage—for backend data management and remote computation. The apps were installed on physical Samsung Galaxy S21 devices (model SM-G991B), equipped with an Exynos 2100 Octa-core processor (1×2.9 GHz Cortex-X1, 3×2.8 GHz Cortex-A78, 4×2.2 GHz Cortex-A55), 8 GB RAM, and running Android 14. Each app operated continuously and silently in the background, collecting five data streams: ambient audio, screenshots, GPS, accelerometer, and gyroscope.

In the Stanford Screenomics platform architecture, each sensor modality was handled by an independent module responsible for local preprocessing and standardization. Collected data were cleaned, deduplicated, compressed, and analyzed for secondary information extraction, then encoded in a uniform timestamped numeric format. Ambient audio was processed to compute minute-level mean decibel values using Android’s AudioRecord API with custom Java/Kotlin signal processing. Screenshots underwent optical character recognition via Google ML Kit’s Text Recognition API, followed by sentiment analysis using an on-device TensorFlow Lite model fine-tuned for sentiment classification (scored 1‐10, the higher indicating more positive sentiment). GPS coordinates triggered real-time weather retrieval through the OpenWeatherMap API, integrated using Retrofit. Motion sensor data (accelerometer and gyroscope) were converted to step counts using Android’s SensorManager API and local step detection algorithms. Real-time phenotyping analysis was performed every 30 minutes. Specifically, the most recent 30 minutes of derived data were held in memory and analyzed using a simple random forest model, implemented via TensorFlow Lite, to identify the single strongest predictor among sentiment, noise, and weather for forecasting physical activity levels (step counts). Cleaned and deduplicated raw media files (audio and screenshots) were compressed with Android’s MediaCodec API and image compression, reduced to ≈10% of original size, and uploaded in batches to Firebase Cloud Storage (for media files such as audio and screenshots) and Firebase Realtime Database (eg, for structured text data such as GPS coordinates and sensor logs). On-device phenotype analysis results were immediately passed to the intervention controller when a newly generated phenotype analysis result differed from the prior one, updating intervention decisions.

By contrast, the traditional architecture used a centralized data collection approach, in which a single node continuously collected raw sensor data without any local preprocessing or secondary information extraction. All collected data were sequentially stored in a single storage, then transmitted to Firebase Cloud Storage (media) and Firebase Realtime Database (text data). Every hour, the most recent 30 minutes of stored data were retrieved from Firebase and processed in a cloud-based computation stage (analogous to cloud 2 in the traditional system architecture), which performs data cleaning, deduplication, and standardization. Secondary information was then extracted within this processing using the same methods as the Stanford Screenomics platform architecture: screenshots were processed using Google ML Kit’s Text Recognition API for optical character recognition, followed by sentiment scoring using a fine-tuned TensorFlow Lite model; audio was processed to compute mean decibel levels via cloud-executed Java signal processing routines; weather scores were retrospectively retrieved by querying the OpenWeatherMap API via HTTP requests for conditions nearest to each GPS timestamp and location coordinate (functionally equivalent to Retrofit calls on Android); and accelerometer and gyroscope data were converted into step counts using reimplemented versions of the Android SensorManager API logic and step detection algorithms, executed in cloud functions. Once all data streams were standardized, phenotype analysis was conducted in the same cloud-based computation stage (cloud 2) using the same random forest model deployed in the Stanford Screenomics platform architecture, identifying the strongest predictor—among sentiment, noise, and weather—of physical activity levels as measured by step counts. In this architecture, the intervention controller was implemented as a separate cloud function (conceptually analogous to cloud 3), which receives phenotype analysis outputs from the computation stage and updates intervention decisions.

### Experiment Setup

Two controlled experiments were conducted to assess the effects of system architecture (traditional vs Stanford Screenomics platform) and varying data load conditions over time on app performance. Both systems were implemented as complete, self-contained applications so that the experiments could evaluate the performance of the architectures holistically (rather than evaluating the various components of each architecture in isolation). Each app was run for 48 hours at four distinct data collection load profiles that differed in the frequency and size of collected sensor data streams—very heavy (≈60 MB/min), heavy (≈40 MB/min), medium (≈30 MB/min), and low (≈10 MB/min). These four load profiles were invoked by using different sampling rates: for very heavy load, one 10-second audio recording was captured per minute alongside sensor data collected every 3 seconds; heavy load involved 7.5-second audio and 5-second intervals for other sensors; medium load used 5-second audio and 8-second sensor intervals; and low load collected 2.5-second audio and 10-second sensor intervals. These load profiles were selected to reflect real-world trade-offs between resolution and processing constraints. In the first experiment, systems ran completely offline to stress-test local device performance, with continuous monitoring of CPU and RAM usage, battery drainage, and data loss events. In the second experiment, the same load conditions were applied over a stable, uninterrupted high-speed Wi-Fi connection to evaluate end-to-end processing latency, with timestamps recorded at key stages of data handling. Data from both experiments are displayed visually and analyzed statistically.

Experiment 1 was designed to directly compare the two architectures in terms of resource efficiency and data completeness under varying load conditions. In this experiment, both systems ran completely offline—without any network connectivity—to stress-test local device performance. Virtual users followed a scripted loop of six everyday smartphone activities, switching tasks every 5 minutes in the following fixed sequence: watching a movie on YouTube, reading eBooks, browsing TikTok, web surfing, listening to Spotify, and scrolling Instagram Reels. Devices were tethered to desktop USB ports supplying 2.5‐5 W power, sufficient to sustain uninterrupted operation while still permitting observable battery depletion. System behavior was continuously monitored under these conditions, with minute-by-minute logging of CPU usage, RAM usage, battery level, and data loss events. Data loss was defined as any failure to capture or store new data due to system overload—caused by excessive CPU usage, RAM usage, or insufficient storage—or failure in secondary data retrieval (eg, null or invalid return values from services).

Experiment 2 repeated these conditions over a 250 Mbps Wi-Fi connection, representative of average US network speeds, to evaluate end-to-end processing latency across both systems. For the Screenomics platform system architecture, five event-based timestamps were recorded per 30-minute processing instance (“trial”): completion of parallel local write operations across all modules; completion of on-device preprocessing and secondary data extraction; parsing and loading of standardized data into memory; completion of phenotype analysis; and delivery of results to the intervention controller. Similarly, for the traditional system architecture, five event-based timestamps were recorded per trial: completion of centralized local write, completion of raw data upload and retrieval of the 30-minute data segment on the cloud for processing, completion of cloud-based preprocessing and secondary data extraction, completion of phenotype analysis, and delivery of results to the cloud-based intervention controller. Although data collection and processing occurred concurrently in both systems, processing timestamps were captured at the completion of each major stage for each 30-minute data window. These timestamps served as logical boundaries, reflecting real-world execution timing where steps are not strictly sequential or isolated.

### Data Analysis

For experiment 1, CPU (%) and RAM usage (MB), battery drainage rate (%/h), and data loss (%) were summarized descriptively across the four load conditions for both the Stanford Screenomics platform and traditional system architectures. The minute-by-minute measurements (2880 observations per load condition) were used to compute means, SDs, and ranges. Differences between architectures were assessed for each load condition using two-sample *t* tests (*P*<.05). CPU and RAM trends were visualized with density plots to assess stability and consistency, where wider distributions indicate greater variability. Data loss was quantified as the proportion of expected sensor data not captured or stored during each one-minute interval, with cumulative trajectories plotted over the 48-hour experiment. Exponential growth rates were calculated to quantify the rate of data loss under each condition.

For experiment 2, the hierarchical structure of the data, with repeated trials—each representing a processing instance initiated every 30 minutes using a 30-minute data segment, during which multiple key-stage timestamps were recorded—nested within each combination of architecture and load condition; differences in total processing time were analyzed using a linear mixed-effects model. As sustained processing can lead to memory accumulation and thermal-induced CPU throttling, later trials were expected to exhibit progressively longer processing times. Accordingly, architecture (Stanford Screenomics platform and the traditional) and load condition (very heavy, heavy, medium, and low) were included as fixed effects, and trial index (ie, sequential processing cycle initiation number) as a covariate to capture temporal effects. This model allowed us to quantify the effects of architecture and load while controlling for systematic variation across sequential trials.

### Ethical Considerations

This study did not involve human participants or identifiable personal data. All experiments consisted of technical evaluations on mobile software and performance metrics derived from scripted simulations. Accordingly, the Stanford University Institutional Review Board confirmed that formal ethical approval was not required for this research.

## Results

**Figure 2.**
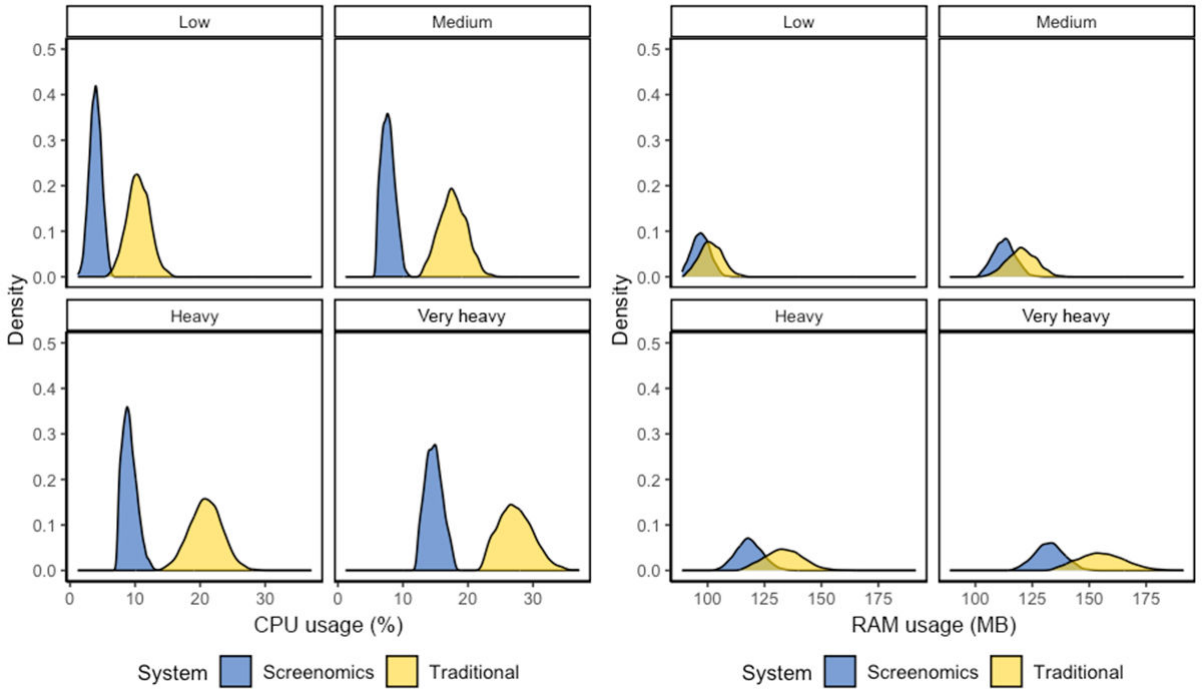
Comparison of the Stanford Screenomics platform system and traditional system resource usage (CPU and RAM) across data load conditions (low, medium, heavy, and very heavy) over the 48-hour experimental period.

**Figure 3.**
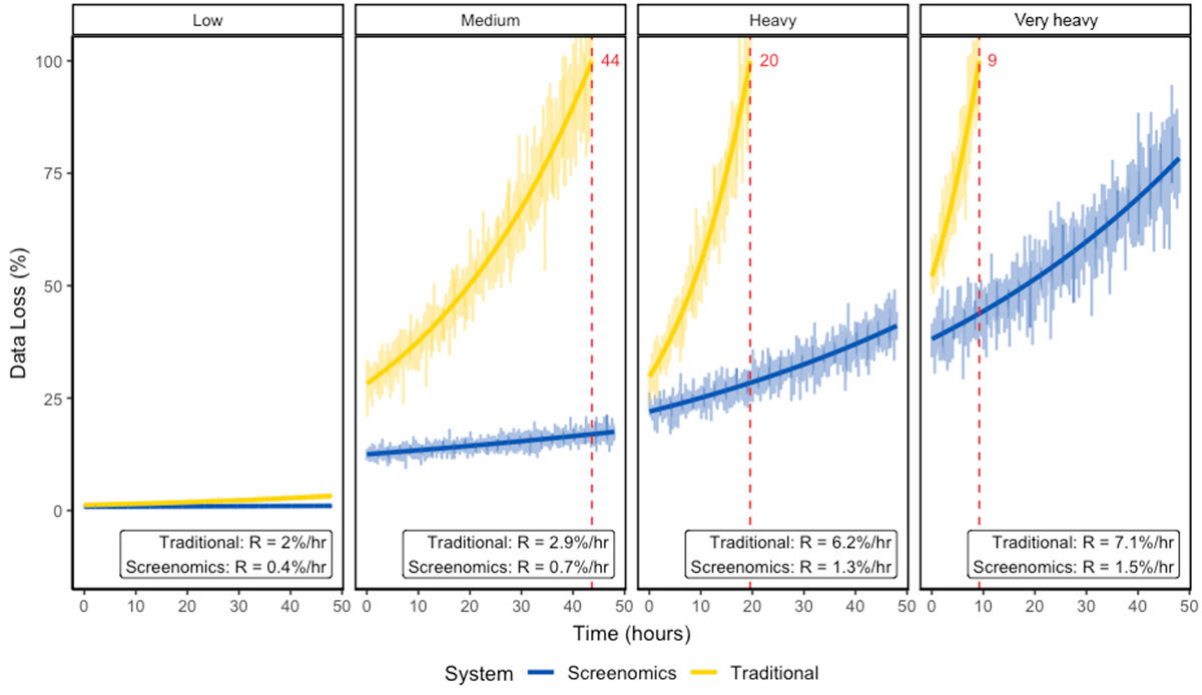
Comparison of the Stanford Screenomics platform system (blue) and traditional system (yellow) data loss across different load levels. Panels show minute-by-minute progression of data loss over time at low, medium, heavy, and very heavy load levels. Each point indicates the proportion of data lost during that one-minute interval (n=2880 per 48-h experiment). The dashed red line denotes the time (in hours) at which the traditional system experienced complete failure, after which there was total data loss (ie, data fidelity=zero).

The results from experiment 1 testing resource efficiency, data completeness, and real-time processing responsiveness under varying load conditions are shown in Table 1 and Figures 2 and 3. As seen in Table 1, both the traditional system architecture and the Stanford Screenomics platform system architecture showed increasing resource demands as data load rose from low to very heavy. As expected, the Stanford Screenomics platform system consistently demonstrated significantly greater efficiency across all metrics and conditions (Table 1 and Figure 2; all *P* values <.001). CPU usage in the traditional system increased from an average of 10.5% (SD 1.7) under low load to 26.9% (SD 2.4) at very heavy load, whereas the Stanford Screenomics platform system maintained substantially lower CPU usage, ranging from 3.9% (SD 0.9) to 14.6% (SD 1.8) across the same conditions. Similarly, RAM usage was significantly lower in the Stanford Screenomics platform system, with memory consumption rising from 97 MB (SD 4.1) under low load to 132 MB (SD 6.6) at very heavy load, compared to 101 MB (SD 5.2) to 155 MB (SD 6.9) in the traditional system. Battery drainage rates also reflected these efficiency gains: the traditional system’s battery depletion increased from 1.4% (SD 0.1) per hour at low load to 3.2% (SD 0.3) per hour under very heavy load, while the Stanford Screenomics platform system consistently consumed less power, ranging from 0.9% (SD 0.1) to 2.1% (SD 0.2) per hour over the same load conditions. Storage growth patterns further highlighted architectural differences: the traditional system exhibited logarithmic growth with backlog accumulation under heavier loads, while the Stanford Screenomics platform pipeline maintained linear storage growth by promptly clearing processed data segments (all storage differences *P*<.001). As CPU, memory, and battery all remained within safe limits during the experiment, the scheduler treated all modules equally throughout the experimental period, with no module being deferred or priority reordered.

**Table 1.** Comparison of Screenomics system and traditional system resource usage and battery drain across data load conditions (low, medium, heavy, and very heavy). Metrics were recorded once per minute over 48 hours (n=2880 per load condition). *P* values indicate statistical significance of differences between the Stanford Screenomics platform system and the traditional system.

Metric and load	Stanford Screenomics platform system		Traditional system		*P* value
	Mean (SD)	Range	Mean (SD)	Range	
CPU usage (%; n=2880)					
Very heavy	14.58 (1.54)	12.14‐18.17	51.93 (3.98)	42.38‐69.11	<.001
Heavy	8.83 (1.34)	7.33‐13.82	22.86 (2.53)	16.02‐29.33	<.001
Medium	7.54 (1.19)	5.95‐11.02	17.61 (2.13)	12.87‐24.59	<.001
Low	3.92 (0.94)	1.05‐6.52	9.51 (1.74)	6.01‐16.65	<.001
RAM usage (MB; n=2880)					
Very heavy	132.34 (6.58)	116.72‐153.03	154.62 (10.88)	134.38‐214.14	<.001
Heavy	118.37 (5.75)	103.57‐142.41	133.19 (8.73)	115.15‐175.93	<.001
Medium	112.61 (4.93)	101.93‐130.83	120.29 (6.38)	103.53‐143.54	<.001
Low	96.99 (4.11)	88.78‐113.57	101.40 (5.16)	90.18‐119.90	<.001
Battery drain (%/h; n=2880)					
Very heavy	2.0 (0.2)	1.8‐2.3	3.2 (0.3)	2.7‐3.7	<.001
Heavy	1.6 (0.2)	1.3‐1.9	2.5 (0.3)	2.1‐3.0	<.001
Medium	1.3 (0.2)	1.1‐1.6	2.0 (0.2)	1.7‐2.4	<.001
Low	0.9 (0.1)	0.8‐1.1	1.4 (0.1)	1.2‐1.6	<.001

Figure 3 illustrates data loss trajectories over 48 hours under the four data load conditions. Across all conditions, the Stanford Screenomics platform system consistently outperformed the traditional system in preserving data fidelity. Under low load, both systems maintained near-complete data capture with minimal or no loss. However, at higher data volumes, differences between the systems became increasingly pronounced. Under medium load, the traditional app failed entirely after approximately 44 hours, whereas the Stanford Screenomics platform sustained continuous data collection for the full duration. Under heavy load, the traditional system failed at around 20 hours, and under very heavy load, it ceased functioning after just 9 hours. In stark contrast, the Stanford Screenomics Platform system remained operational throughout all 48 hours under every load condition, exhibiting only a gradual increase in data loss over time and never reaching complete failure.

As seen in the figure, data loss trajectories followed an exponential growth pattern, with the steepness of each curve reflecting both data volume and the system’s capacity to manage resource constraints. This pattern aligns with compounding effects in CPU and RAM usage: as processing delays mount, the likelihood of subsequent data loss increases, accelerating cumulative degradation. Under very heavy load, the traditional system exhibited the fastest failure trajectory, with a growth rate (R) of 7.1% per hour. In comparison, the Stanford Screenomics platform system maintained a much lower growth rate of 1.5% per hour. Similar disparities were observed under other conditions: at heavy load, the traditional system grew at 6.2% per hour vs 1.3% for the Stanford Screenomics platform; at medium load, 2.9% vs 0.7%; and at low load, 2% vs 0.4%. These patterns reflect the traditional system’s vulnerability to frequent CPU spikes and RAM saturation, resulting in sharp inflection points in cumulative loss. The exponential curves for the traditional system suggest that once resource bottlenecks emerge, loss accelerates rapidly. In contrast, the Stanford Screenomics platform architecture produced shallower, more linear curves, reflecting the architecture’s ability to maintain real-time processing and proactively manage memory, thereby preventing cascading failure even under substantial load. By the end of the 48-hour data collection period, the traditional system captured only 4.7%, 17.2%, 39.4%, and 97.9% of the expected data under very heavy, heavy, medium, and low load conditions, respectively. In contrast, the Stanford Screenomics platform system captured 44.2%, 69.5%, 85.2%, and 99.1%, demonstrating up to 9.4 times greater data fidelity under the highest load.

**Figure 4.**
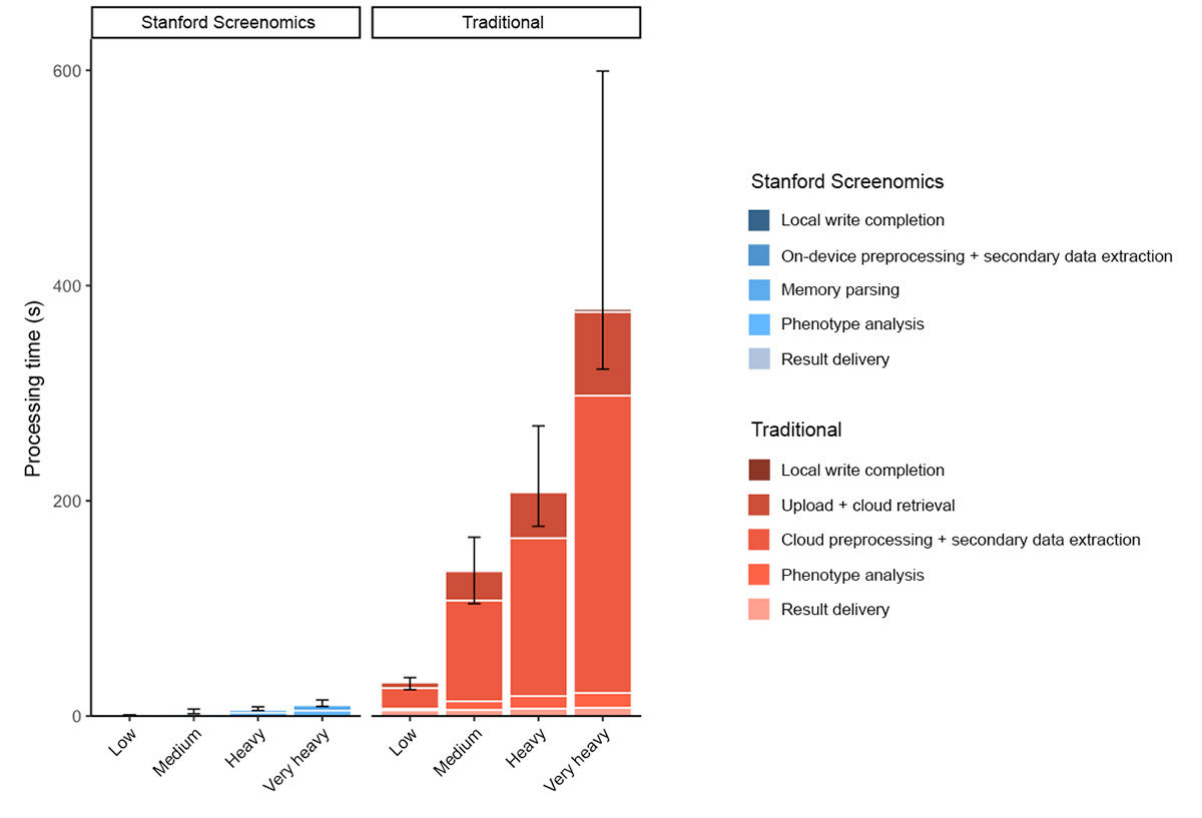
Comparison of the Stanford Screenomics platform system and traditional system median end-to-end processing time across data load conditions.

The results from experiment 2 testing architecture differences in total processing time under the four load conditions and over time are shown in Table 2 and Figure 4. The linear mixed-effects model revealed significant main effects of system architecture, load condition, and trial index (all *P*<.01), indicating that processing times increased over repeated trials due to cumulative device burden, with the Stanford Screenomics platform consistently outperforming the traditional system across all load levels.

**Table 2.** Comparison of the Stanford Screenomics platform system and traditional system end-to-end processing time across data load conditions. Metrics correspond to 96 processing instances (“trials”) per load condition. Trials were initiated every 30 minutes and used a 30-minute data segment, during which multiple key-stage timestamps were recorded to capture completion times for major processing steps.

Processing step	Low (n=96)	Medium (n=96)	Heavy (n=96)	Very heavy (n=96)
Stanford Screenomics platform system (s, median [IQR])				
Local write completion	0.001 (0.001‐0.001)	0.004 (0.003‐0.005)	0.005 (0.004‐0.007)	0.010 (0.007‐0.013)
On-device preprocessing + secondary data extraction	0.090 (0.075‐0.122)	0.452 (0.361‐0.584)	0.704 (0.553‐0.982)	1.281 (1.09‐1.628)
Memory parsing	0.342 (0.283‐0.431)	1.654 (1.006‐2.703)	2.451 (2.083‐3.245)	5 (4.012‐5.680)
Phenotype analysis	0.444 (0.374‐0.43)	1.823 (0.78‐2.72)	3.1 (2.32‐3.90)	5.0 (3.87‐7.35)
Result delivery	0.018 (0.012‐0.025)	0.022 (0.013‐0.027)	0.027 (0.015‐0.037)	0.032 (0.019‐0.046)
Total processing time	0.895 (0.722‐1.021)	3.955 (2.124‐6.484)	6.287 (5.171‐8.554)	9.323 (8.94‐14.957)
Traditional system (s, median [IQR])				
Local write completion	0.027 (0.020‐0.034)	0.144 (0.115‐0.188)	1.221 (0.172‐1.985)	2.883 (0.313‐10.541)
Upload + cloud retrieval	5.343 (4.133‐6.492)	27.245 (21.0‐33.4)	42.752 (34.085‐52.247)	77.64 (61.534‐102.325)
Cloud preprocessing + secondary data extraction	18.933 (15.537‐22.012)	93.585 (74.403‐114.151)	146.492 (118.582‐178.19)	276.194 (220.637‐333.321)
Phenotype analysis	1.565 (1.225‐1.963)	8.138 (6.432‐10.520)	11.75 (10.331‐15.656)	13.863 (11.248‐29.834)
Result delivery	5.24 (4.796‐5.776)	5.53 (5.02‐6.154)	6.769 (6.099‐7.656)	7.53 (6.552‐18.532)
Total processing time	30.108 (24.515‐35.672)	134.642 (104.4‐166.093)	229.984 (176.269‐269.584)	398.11 (322.281‐599.233)

Table 2 summarizes the median (IQR) end-to-end processing times for both systems across four data load conditions. Under low load, the Stanford Screenomics platform system completed a full phenotype update in 0.90 (IQR 0.72‐1.02) seconds, compared to 30.1 (IQR 24.5‐35.7) seconds for the traditional system. As data load increased, the Stanford Screenomics platform maintained low processing times, with median durations of 3.96 (IQR 2.12‐6.48) seconds, 6.29 (IQR 5.17‐8.55) seconds, and 9.32 (IQR 8.94‐14.96) seconds for medium, heavy, and very heavy loads, respectively. In contrast, the traditional system exhibited progressively longer processing times: 134.6 (IQR 104.4‐166.1) seconds under medium load, 230.0 (IQR 176.3‐269.6) seconds under heavy load, and 398.1 (IQR 322.3‐599.2) seconds under very heavy load.

The most substantial latencies in the traditional system were attributed to cloud preprocessing and secondary data extraction, which reached a median of 276.2 (IQR 220.6-333.3) seconds under very heavy load (Table 2). Combined with delays from cloud upload and retrieval, these steps accounted for the majority of end-to-end processing time. In the Stanford Screenomics platform system, memory parsing and phenotype computation contributed the most to processing duration, particularly under higher loads, though total processing time remained within real-time boundaries across all conditions. Across the different data volumes, the Stanford Screenomics platform achieved approximately 34 to 43 times faster total processing than the traditional system.

In addition to faster median processing times, the Stanford Screenomics platform system demonstrated markedly more stable performance across all data load conditions (Table 2 and Figure 2). IQRs remained narrow, particularly under low and medium data loads, indicating consistent processing times with minimal variability. The Stanford Screenomics platform system’s IQRs expanded only slightly from 0.3 seconds under low load to 6 seconds under very heavy load. In contrast, the traditional system exhibited substantial and growing variability as data volume increased, with IQRs expanding from 11 seconds under low load to nearly 5 minutes under very heavy load. This instability likely reflects compounded delays from cloud upload, retrieval, and server-side queuing, underscoring the limitations of cloud-dependent architectures for real-time phenotyping.

## Discussion

### Principal Findings

Digital phenotyping is increasingly being used to support health monitoring, clinical assessment, risk detection, and adaptive intervention delivery. To date, much of this progress has been enabled by traditional cloud-centric architectures that support large-scale longitudinal monitoring, retrospective behavioral analysis, population-level risk stratification, and the development of many foundational digital phenotypes. These systems remain well suited for applications in which phenotype generation can occur over extended time horizons, such as epidemiologic research and treatment survival surveillance. However, as digital health tools transition from small proof-of-concept studies to large-scale, long-term clinical deployments, the ability to generate accurate and timely digital phenotypes becomes increasingly critical for patient safety and treatment efficacy. Emerging applications—including just-in-time adaptive interventions, relapse prevention, acute stress detection, and context-aware behavioral coaching—require phenotype updates that closely reflect an individual’s current state. Existing architectures commonly rely on sequential, cloud-dependent processing workflows that are vulnerable to network latency, data loss, and service interruptions, which can compromise the timeliness and completeness of digital phenotype generation. To address these challenges, we developed, implemented, and evaluated the Stanford Screenomics platform architecture, a modular framework combining parallel processing, early-stage multimodal data standardization, and edge (on-device) computation. Experimental results demonstrated substantially improved computational efficacy, reliability, and responsiveness relative to the traditional sequential, cloud-based architecture being used in current systems. By supporting real-time phenotype generation while maintaining longitudinal analytic capabilities, the Stanford Screenomics platform has the potential to extend the utility of digital phenotyping beyond retrospective monitoring and improve both the scalability and clinical viability of smartphone-based digital phenotyping.

### Early-Phase Standardization and Edge Computation

The Stanford Screenomics platform architecture advances mobile digital phenotyping by eliminating data transmission delays, making real-time phenotyping feasible on standard consumer smartphones. The core innovation lies in a tightly integrated two-layer architecture—comprising a data collection layer and a data management layer—designed to operate in tandem for low-latency, clinically actionable analysis. While traditional systems defer transformation until after raw data are uploaded to the cloud, our data collection layer implements an early-phase standardization framework that resolves data heterogeneity directly at the source. Although the concept of metadata-guided early-phase standardization has been suggested previously, prior mHealth systems were unable to realize it effectively due to technical and practical challenges [41-44]: early smartphones lacked the capability to process multiple real-time data streams [13,45], and building robust standardization frameworks required substantial technical infrastructure and development resources [46,47]. Furthermore, clinical risk aversion and established workflows historically favored centralized, postcollection cloud processing, resulting in widespread reliance on simpler but less responsive methods [48-50]. Traditional systems have thus introduced significant delays, particularly under heavy data loads. Delays of even a few minutes can result in phenotype estimates that no longer reflect a patient’s current behavioral or medical state. Now, leveraging advances in smartphone technology and the associated software development tools, the Stanford Screenomics platform architecture opens the possibility to resolve heterogeneity at the source, obtain standardized outputs in real time, and eliminate the need for downstream parsing and formatting, thereby reducing computational complexity and speeding up phenotyping.

In this new architecture, the data management layer leverages edge computation to facilitate real-time phenotype analysis without the overhead of disk I/O and network delays. Incoming data streams are continuously evaluated in memory to identify complex, context-specific individual-level phenotypic traits, such as prolonged sedentary behavior when a patient is alone during adverse weather. The intervention controller then consumes these dynamic phenotype outputs to generate continuous, moment-by-moment intervention decisions tailored to the individual’s current state. This localized computation further enhances energy efficiency and fault tolerance by isolating processing to temporary data segments [13,19,20]. Together, early-phase standardization and in-memory processing form a scalable, resilient pipeline optimized for real-world phenotyping on mobile devices. In our experiment 1, the Stanford Screenomics platform architecture consistently achieved 34- to 57-fold faster end-to-end processing than a traditional cloud-based architecture, maintaining total processing times from data acquisition to phenotype update below 10 seconds across all data load conditions. These findings affirm that reducing cloud dependency—by shifting transformation and analysis to the device—is a critical design principle that supports high-performance, real-time phenotyping on smartphones, with potential applications for proactive adaptive interventions, real-time alerts, and interactive individual monitoring.

### Modular Architecture and Parallel Processing

Another critical strength of the Stanford Screenomics platform architecture is its modular design with distributed file storage, which together enable parallel data collection and processing throughout the entire pipeline. By allowing each data node to operate independently, the system avoids the storage contention and blocking common to the centralized, sequential writes found in traditional designs [51]. The modularity enhances throughput, prevents delays caused by sequential data handling, and allows for efficient resource management and reliable data handling on consumer devices, particularly under heavy data loads [7,17,18]. The results from experiment 1 validate the operational reliability of this approach: the Stanford Screenomics platform architecture maintained uninterrupted data capture for 48 hours across all data load levels, while the traditional architecture failed between 44 and 9 hours in medium to very heavy load conditions. These accumulated failures followed exponential data loss trajectories alongside rising device CPU and memory usage, leading to severe performance instability under high data loads. This contrast underscores the clinical risk of traditional architectures under stress, whereas our modular, memory-aware design mitigated cascading degradation. Narrow statistical variances (IQRs) in processing times further demonstrate our system’s stability and consistency, whereas the traditional system’s unpredictable performance could lead to critical gaps in patient monitoring. This level of technical reliability is especially crucial for real-world clinical deployments where patient internet connectivity is often limited, unstable, or intermittent. When a stable network is available, data are regularly uploaded and deleted from the device to free storage space. However, during offline periods, data accumulate locally and cannot be offloaded, increasing vulnerability to storage and processing failures. Any local capture failure under these conditions leads to permanent, unrecoverable data loss. Therefore, the ability to securely store and process data entirely on-device—without relying on network availability—is essential for supporting continuous phenotyping, long-term monitoring, and timely interventions.

### Limitations, Considerations and Outlook

Although the experiments benchmarked the Stanford Screenomics platform architecture against a traditional architecture under controlled conditions, several limitations warrant consideration. This study relied on scripted virtual users (5 min per task) and fixed device and network configurations. This experimental setup enabled precise measurement but does not fully capture real-world variability, such as highly idiosyncratic individuals’ multitasking and app switching behaviors, smartphone models, OS versions, memory capacities, background processes, or battery health. Experiments were limited to 48 hours, which was sufficient for evaluating short-term device burden and revealing key architectural differences. Longer-term deployments could reveal cumulative effects on memory management, thermal performance, battery longevity, and overall system stability—effects we expect to be even more favorable for the Stanford Screenomics platform architecture, as its dynamic task scheduler increasingly optimizes performance over time. Network conditions were idealized with stable, high-speed Wi-Fi; in real-world scenarios, individuals frequently encounter cellular dead zones or intermittent connectivity, which may introduce latency, data loss, or integrity issues that disrupt continuous care [52,53]. The current study focused on a limited set of data streams with fixed sampling rates, whereas nonfixed sampling—such as event-based, random, or context-triggered data, including user-smartphone interactions or physiological signals—could impose unpredictable demands on device processing [54,55]. The outcome metrics focused primarily on system-level performance (CPU/RAM usage, battery drain, and storage use); however, operational outcomes directly affecting individual experience—such as app responsiveness, intervention timing, and the downstream impact of partial or corrupted data on phenotype accuracy—were not assessed. Data loss measured at discrete points may underestimate the cascading consequences on real-time processing or the timely delivery of critical health interventions. Importantly, this study evaluated the computational architecture underlying digital phenotyping rather than the validity or clinical utility of the resulting phenotypes. Future work should examine whether these architectural improvements translate into more accurate and robust phenotype generation and improved downstream clinical outcomes in real-world deployments. In sum, future studies should extend evaluations to longer-term real-world deployments across diverse devices, OSs, and network conditions, incorporate additional high-bandwidth or continuous streams, and assess operational outcomes to better understand practical performance and guide the development of adaptive strategies for reliable, scalable digital phenotyping.

We also note some critical operational considerations surrounding the deployment of parallel processing and edge computing in mHealth systems. Parallel modules improve throughput but can introduce latency and reduce real-time responsiveness when threads wait for shared resources or for other modules to complete their processing window [56,57]. This issue is common in mobile parallel computing, particularly with high-frequency or high-volume data streams. The Stanford Screenomics platform mitigated most locking and buffering problems by isolating preprocessing within each module, sending only small, standardized outputs to shared memory, and performing lightweight in-memory fusion, minimizing thread contention and OS-induced delays. While these hardware limitations cannot be entirely eliminated, careful pipeline design—such as module-level preprocessing, data conversion or compression, and in-memory analysis—can make their impact negligible. Another consideration is diminishing returns, as described by Amdahl’s law, where overall system speedup is limited by the portions of the code that cannot be parallelized [58]. In our platform, this limitation was not observed because the data pipeline was highly parallelizable across all stages—data collection, preprocessing, storage, and memory load—with each sensor processed independently. Potential bottlenecks, such as writing aggregated data to storage, were largely avoided through distributed module-level storage. By contrast, even a highly parallelized system that relies on centralized storage, as in traditional sequential architectures, can experience contention, limiting overall throughput [59-61]. Developers of mHealth systems should therefore maximize parallelism across all stages, identifying and optimizing nonparallelizable components (such as steps requiring synchronized multisensor streams during multimodal feature extraction) and exploiting often-overlooked opportunities such as preprocessing, distributed storage, and memory load [62-64]. In such cases, best practices include incremental or rolling computation, asynchronous buffering, prioritizing lightweight operations for time-sensitive tasks, decoupling pipelines to isolate sequential fusion steps, and optimizing nonparallelizable algorithms to minimize latency. The shared Stanford Screenomics platform’s reference architecture codebase implements these strategies, with structured cross-references throughout to help digital health researchers trace how each best practice is realized.

Edge computing enables real-time, on-device analytics for mobile digital phenotyping but comes with trade-offs [25,65]. High-complexity algorithms—such as deep neural networks for psychiatric risk prediction, temporal sequence models, or advanced multimodal fusion—can easily exceed standard smartphone capacity, particularly when multiple high-frequency streams are active simultaneously. Long monitoring windows or large-scale temporal modeling can increase device processing demands. Mobile sensor streams are notoriously prone to data missingness, noise, and misalignment, which can distort clinical feature extraction and reduce diagnostic accuracy if not handled robustly. Real-time, in-memory analyses often permit only incremental updates rather than full model retraining or comprehensive statistical evaluation, limiting interpretability and the detection of subtle or long-term trends, which requires careful balancing between temporal fidelity, model complexity, and available device resources. To mitigate these constraints without compromising clinical utility, several optimization strategies can be implemented: lightweight, incremental, or rolling-window feature extraction reduces memory footprint while preserving temporal fidelity; simplified or approximate fusion methods prioritize critical modalities without overtaxing the device; robust preprocessing, interpolation, and confidence thresholds handle noisy or incomplete streams; and adaptive scheduling or offloading of noncritical tasks during idle periods balances computational load [66,67]. As a recommended strategy for future implementations, investigators could use a hybrid approach: lightweight proxy models run continuously on-device to process critical streams and trigger immediate, time-sensitive interventions, while heavier, more complex models run intermittently on buffered data to update the proxy’s parameters and maintain predictive alignment. Cloud processing can further support large-scale, longitudinal, or multiparticipant population-level analyses that exceed on-device capacity, enabling more complex epidemiologic modeling, long-term trend detection, and cross-user insights. In the Stanford Screenomics platform, portions of preprocessed data are temporarily loaded into memory and discarded afterward to reduce memory pressure and support continuous responsiveness, and standardized module outputs produce small, uniform representations that simplify computation, minimize thread contention, and facilitate multimodal fusion. Future systems could extend efficiency through dynamic buffer resizing, prioritized caching, shared memory pooling, lightweight streaming compression, and memory-aware sampling, while checkpointing with rollback, incremental cleanup, and adaptive model selection can ensure memory usage remains bounded under variable workloads. By combining these strategies, investigators can preserve real-time responsiveness, leverage advanced diagnostic models, and maintain device performance within safe limits, enabling adaptive, on-device digital phenotyping without overloading the device or relying on cloud computation.

### Conclusions

The Stanford Screenomics platform architecture resolves the chronic latency and reliability issues of traditional sequential, cloud-based digital phenotyping by adopting a modular architecture design that successfully integrates parallel processing with edge computing. By synergizing resource monitoring, adaptive task scheduling, metadata-guided multimodal data fusion, distributed storage, and in-memory caching, this framework shifts high-intensity computation directly to the mobile device, maintaining high data fidelity even during network outages. This study contributes the field’s first validated software reference architecture for smartphone-based real-time digital phenotyping, providing a standardized blueprint that overcomes the fundamental engineering hurdle of resource management on constrained hardware. By releasing the validated prototype architecture as an open-source reference, this work allows digital health researchers and clinical trialists to bypass the steep engineering barriers of data synchronization and system instability. Instead, investigators can focus their resources on high-level phenotype discovery and validation. Ultimately, this platform establishes a highly resilient, scalable foundation to support the next generation of reliable, context-aware digital health interventions deployed directly on patients’ mobile devices.

## Supplementary material

10.2196/87320 Multimedia Appendix 1 The Stanford Screenomics platform architectural components.

## Abbreviations

mHealth: mobile health
