# Supplementary material for: Software Reference Architecture for Real-Time Mobile Digital Phenotyping: Evaluation of System Designs
Source: JMIR Form Res. 2026 Aug 7;10:e87320. doi: 10.2196/87320 (PMC13451010; doi:10.2196/87320)
Supplement: Supplementary file 1 [file formative-v10-e87320-s001.docx]

**Note S1.**

***The Stanford Screenomics Platform Architectural Components***

The Stanford Screenomics platform, shown on the left side of **Figure 1**, features a modular architecture where the data processing pipeline is structured in two layers—(a) *data collection layer* with a *unified fusion standard* (e.g., data structure, preprocessing steps, metadata conventions), and (b) *data management layer* equipped with a *distributed file storage system* and *intervention controller*. *Parallel computing* is employed throughout. Separate modules manage each data source and invoke data fusion processes, producing fused data support real-time on-device phenotype updates, which feed the intervention controller to generate personalized, adaptive interventions. Each incoming data is cached and treated as a task; initially, all tasks are prioritized equally for real-time phenotype updates. Modules report their current load and processing status to a task scheduler that dynamically adjusts parallelism across modules. When system resources approach thresholds (CPU > 85%, memory < 200 MB, battery < 20%), the scheduler executes lighter modules first, deferring heavier ones to prevent overload.

The data collection layer consists of *data nodes* and *adapters*, which together provide a unified interface for handling heterogeneous data sources. Each data node is attached to a module, captures raw input from a specific source—such as a device sensor, system broadcast, or user interaction, and emits this data in a structured but minimally processed format. The adapters then apply a series of transformations, including cleaning, de-duplication, compression, and standardization, in accordance with modality-specific transformation rules. These rules are guided by two key metadata components: the data description, which ensures traceability by recording metadata such as the data source, acquisition method, and timing; and the data entity, which defines the modality-specific structure of the desired data object, including its attributes, types, value ranges, and relationships with other data entities. Adapters can also enrich the cleaned data by extracting or retrieving contextually relevant secondary information required for downstream analysis. For example, the GPS adapter can query local weather; the audio adapter may estimate environmental noise; and the screenshot adapter can process text content using optical character recognition (OCR) for in-process sentiment analysis. These enriched streams are emitted as distinct but linked outputs.

The data management layer comprises *a distributed file storage system* and *an edge computation system* supported by in-memory caching mechanisms. The distributed file storage system efficiently manages the massive data streams flowing from each module’s data collection layer, providing high-throughput ingestion, fault tolerance, and rapid retrieval. Incoming data are batched for cloud transmission while simultaneously being selectively cached into memory for immediate edge computing. Within memory, small data subsets are temporally aligned into analyzable units, enabling real-time phenotype analysis (i.e., dynamic DP) and continuous updating of an individual’s phenotype based on momentary changes in environmental, physiological, or behavioral states. This resulting phenotype represents the individual’s multivariate state at that specific moment and is promptly forwarded to the intervention controller. Meanwhile, the complete raw datasets, transferred in parallel to the cloud where they are stored for offline computation, large-scale analyses (i.e., longitudinal DP), or other scheduled cloud-based tasks.

Within each module, in-memory caching is tailored to specific data types and size constraints. Cache management incorporates lifecycle awareness to clear or persist data based on configurable time intervals defined by phenotype analysis requirements, thereby ensuring data consistency and relevance. Additionally, asynchronous data loading and prefetching strategies enable modules to prepare and align data for immediate processing. For example, pre-processed data from the past 30 minutes may be cached and synchronized to maintain temporal alignment across streams, while older data are discarded.

In scenarios requiring rapid feedback—such as detecting acute stress, abrupt behavioral shifts, or deviations in physiological patterns—data must be processed with minimal latency. In-memory analysis bypasses delays associated with disk I/O or cloud transmission by leveraging the device’s RAM to perform fast, local computations. This enables continuous, moment-by-moment alignment and transformation of sensor streams to extract context-relevant features in real time. For example, local weather and media sentiment can be combined with recent activity patterns to detect early signs of sedentary behavior, prompting timely on-device interventions. This approach supports ***dynamic digital phenotyping***, in which the individual's phenotype is continuously refined in real time to reflect their evolving state and context.

In scenarios where immediate responsiveness is not critical—such as monitoring long-term health trends, generating clinical insights, or personalizing treatment strategies—data can be processed remotely using cloud-based analysis pipelines. These pipelines are typically deployed on cloud servers or virtual machines and are optimized for handling large, longitudinal datasets. This cloud-based mode of analysis enables integration of weeks or months of multimodal data, allowing for more computationally intensive processing, such as temporal modeling, feature selection, or the training of predictive models. For example, long-term records of physical activity, screen usage, mood reports, and environmental exposure can be aggregated and analyzed to identify stable patterns associated with behavior change, relapse risk, or treatment response. This cloud-based approach supports ***longitudinal digital phenotyping***, in which phenotypes are constructed from historical data to reveal slow-changing or cumulative traits.

The **intervention controller** is responsible for real-time, context-aware decision making based on the continuously updated momentary digital phenotype generated by the data management layer. This module evaluates whether an intervention is warranted at a specific moment, and if so, selects the most appropriate type, timing, and delivery strategy. By integrating dynamic information—such as behavioral patterns, physiological signals, and environmental context—the module enables personalized, adaptive interventions. For instance, if a typically active user becomes sedentary during a rainy day, the controller may issue a motivational prompt to encourage indoor physical activity. Intervention controllers can vary widely in their complexity depending on the application’s goals and technical requirements [43–46]. Simpler controllers may be static rule-based, issuing alerts or reports based on predefined thresholds, or monitoring-based, continuously tracking phenotypic changes to detect acute deviations. More advanced designs employ adaptive rule-based or knowledge-based approaches, applying machine learning or data mining techniques to uncover patterns and associations. At the most sophisticated level, prediction-based controllers forecast future states or intervention outcomes, enabling *proactive decision making* [46–49]. For example, such a system might simulate how a person is likely to respond to different intervention strategies based on their evolving phenotype profile.

***Traditional Architectural Components***

The Traditional digital phenotyping architecture, shown on the right side of Figure 1, adopts a centralized, cloud-dependent design with a sequential processing pipeline, often spanning up to three separate cloud locations. This traditional approach features a single-layer architecture focused solely on data collection, as all downstream operations—including preprocessing, fusion, analysis, and intervention—are deferred to cloud servers. Multiple sensor data streams collected by a single node are written sequentially to a shared central storage. When multiple streams generate data simultaneously (e.g., synchronized samples at the 00th second), the node must queue writes, waiting for one stream to finish before writing the next to storage. This often introduces delays already at the point of collection. Once stored, raw data are transmitted to Cloud 1, which primarily performs basic staging and filtering to organize the incoming streams. After sufficient data have accumulated (typically based on a fixed time window), the staged data are filtered and streamed to Cloud 2 where extensive preprocessing, multimodal data fusion and analysis occur. Unlike modular systems, data handling in this architecture is tightly coupled, with little separation between preprocessing, fusion, and analysis. For large datasets, this inter-cloud transfer introduces considerable latency. Phenotype analysis—if conducted—only occurs after all the upstream steps are completed.

Because the entire pipeline depends on full data transmission, filtering, and processing before analysis, real-time phenotype profiling is effectively infeasible. If interventions are supported, they are typically triggered from Cloud 3 via cloud notifications based on delayed outputs from Cloud 2. Consequently, the intervention controller used in this architecture is thus often static rule-based, limited to *reactive decision making*, and does not have real-time adaptive capability. The architecture’s heavy reliance on cloud infrastructure significantly constrains responsiveness, scalability, and robustness.

While sequential, cloud-centric architectures remain the most widely adopted approach in digital phenotyping, several alternative designs have been proposed, including hybrid cloud–edge and fog-assisted architectures [50,51]. These approaches move portions of preprocessing or analysis closer to the device, reducing transmission requirements and improving responsiveness. However, these alternative designs remain application-specific and typically lack standardized multimodal fusion frameworks, integrated intervention controllers, dynamic resource scheduling, or unified metadata standards. Consequently, fully integrated architectures supporting simultaneous real-time and longitudinal digital phenotyping remain rare in practice [52].
